# deltaTE: Detection of Translationally Regulated Genes by Integrative Analysis of Ribo‐seq and RNA‐seq Data

**DOI:** 10.1002/cpmb.108

**Published:** 2019-10-17

**Authors:** Sonia Chothani, Eleonora Adami, John F. Ouyang, Sivakumar Viswanathan, Norbert Hubner, Stuart A. Cook, Sebastian Schafer, Owen J. L. Rackham

**Affiliations:** ^1^ Program in Cardiovascular and Metabolic Disorders Duke‐NUS Medical School Singapore; ^2^ Cardiovascular and Metabolic Sciences Max Delbrück Center for Molecular Medicine in the Helmholtz Association (MDC) Berlin Germany; ^3^ DZHK (German Centre for Cardiovascular Research) partner site Berlin Germany; ^4^ Charité‐Universitätsmedizin Berlin Germany; ^5^ National Heart Centre Singapore Singapore; ^6^ National Heart and Lung Institute Imperial College London London U.K.; ^7^ MRC‐London Institute of Medical Sciences Hammersmith Hospital Campus London U.K.

**Keywords:** deltaTE, Ribo‐seq, RNA‐seq, translation efficiency, translational regulation

## Abstract

Ribosome profiling quantifies the genome‐wide ribosome occupancy of transcripts. With the integration of matched RNA sequencing data, the translation efficiency (TE) of genes can be calculated to reveal translational regulation. This layer of gene‐expression regulation is otherwise difficult to assess on a global scale and generally not well understood in the context of human disease. Current statistical methods to calculate differences in TE have low accuracy, cannot accommodate complex experimental designs or confounding factors, and do not categorize genes into buffered, intensified, or exclusively translationally regulated genes. This article outlines a method [referred to as deltaTE (ΔTE), standing for change in TE] to identify translationally regulated genes, which addresses the shortcomings of previous methods. In an extensive benchmarking analysis, ΔTE outperforms all methods tested. Furthermore, applying ΔTE on data from human primary cells allows detection of substantially more translationally regulated genes, providing a clearer understanding of translational regulation in pathogenic processes. In this article, we describe protocols for data preparation, normalization, analysis, and visualization, starting from raw sequencing files. © 2019 The Authors.

**Basic Protocol**: One‐step detection and classification of differential translation efficiency genes using DTEG.R

**Alternate Protocol**: Step‐wise detection and classification of differential translation efficiency genes using R

**Support Protocol**: Workflow from raw data to read counts

## INTRODUCTION

Next‐generation sequencing methods have become commonplace tools in the life sciences, allowing researchers to understand the molecular mechanisms underpinning cellular processes, shaping phenotypic differences, and ultimately modifying disease susceptibility. While it is evident that mining every layer of gene expression would be required for a thorough understanding of gene regulation, expression profiling studies most commonly focus on the abundance of RNA molecules.

RNA sequencing (RNA‐seq) is a methodology that quantifies fragments of RNA molecules to assess the level of gene transcription. To achieve this, sequencing reads are mapped to the genome and counted to quantify the expression of each gene. Significant changes in these counts between conditions identify genes undergoing transcriptional regulation. However, RNA‐seq alone does not capture the full picture. While transcription serves to generate a broad collection of transcripts, the final expression of a gene is refined, and its fate determined, in the downstream stages of gene expression regulation, such as translational regulation, protein stability, protein degradation, and others.

**Figure 1:**
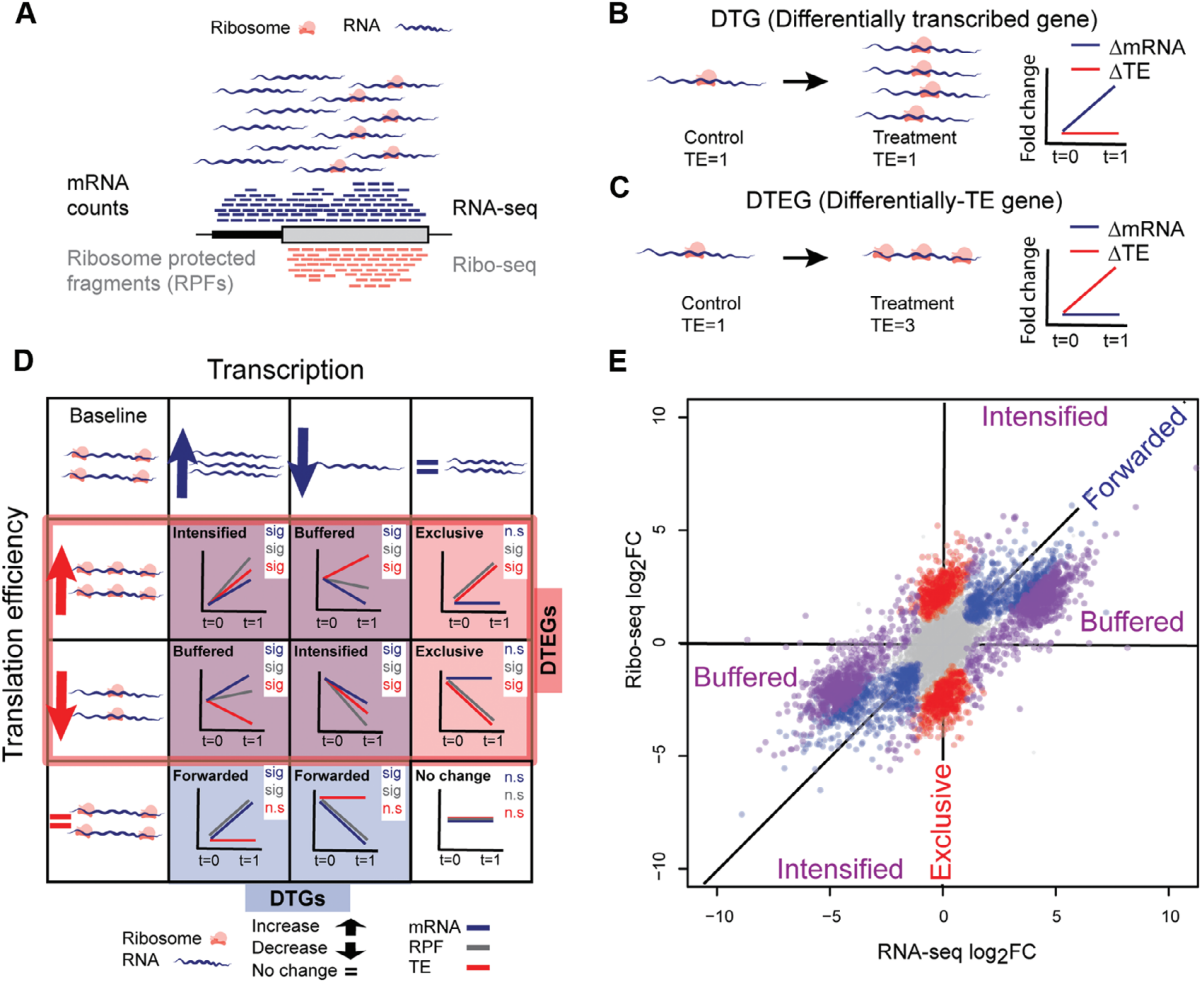
Transcriptional and translational regulation (**A**). Genome‐wide quantification of mRNA counts and ribosome‐protected mRNA fragments (RPFs) using RNA sequencing (RNA‐seq) and ribosome profiling (Ribo‐seq), respectively. Lines are not drawn to scale. In a hypothetical study with two conditions, control and treatment, (**B**) a gene with change in mRNA counts and RPFs at the same rate is a differentially transcribed gene (DTG) and, (**C**) a gene with change in RPFs independent of change in mRNA counts, which leads to a change in translation efficiency, is defined as a differential translation efficiency gene (DTEG). TE = translation efficiency = RPF/mRNA. (**D**‐**E**) Classification of genes based on fold changes of RPF, mRNA, and TE. (D) A gene could be either/both DTG and/or DTEG, and based on the direction of change would fall into one of the eight gene‐regulatory possibilities (sig: significant, n.s.: not significant). Translationally forwarded genes are DTGs that have a significant change in mRNA and RPF at the same rate, with no significant change in TE. Conversely, translationally exclusive genes are DTEGs that have a significant change in RPF, with no change in mRNA leading to a significant change in TE. Several genes are both DTGs and DTEGs, and their regulatory class is determined based on a combination of the relative direction of change between transcription and translation efficiency. Specifically, translationally buffered genes have a significant change in TE that counteracts the change in RNA; hence, buffering the effect of transcription. Translationally intensified genes have a significant change in TE that acts with the effect of transcription. In all cases, the change in RNA can be either positive or negative, and where buffering or intensifying takes place, the direction of change is taken into account. For example, a gene that exhibits an increase in transcription and an increase in translation efficiency is classified as intensified, while a gene that exhibits an increase in transcription but a decrease in translational efficiency is classified as buffered. (E) Simulated data showing fold changes for each gene in RNA‐seq and Ribo‐seq data. Translationally forwarded genes (in blue), exclusive genes (in red), buffered genes (in purple), and intensified genes (in purple) are highlighted.

Ribosome profiling (Ribo‐seq) offers a quantitative approach to study translational regulation, a post‐transcriptional process affecting protein levels. Transcriptome‐wide translation is quantified via the capture of ribosome‐protected RNA fragments (RPFs; Ingolia, Ghaemmaghami, Newman, & Weissman, 2009; also see Fig. 1A). Changes in the number of RPFs between conditions for a given gene can be used as a proxy for a change in the translation of the encoded protein. However, reliably identifying differences in translational regulation is complicated by the fact that the mRNA abundance of the transcript directly affects the probability of ribosome occupancy.

The number of ribosomes per transcript can be estimated by integrating RNA‐seq and Ribo‐seq to calculate translation efficiency (TE), the ratio of the RPFs over mRNA counts within a gene's coding sequence (CDS). TE is essentially the number of ribosomes per gene, normalized to transcript abundance. Genes with changes in TE between conditions are considered to undergo translational regulation [differential translation efficiency genes (DTEGs)]. Specifically, a gene is classified as DTEG if the changes in the number of RPFs cannot be explained by variation in mRNA read counts. A gene with a significant change in its mRNA counts and a concordant change in RPFs is transcriptionally, but not translationally, regulated [differentially transcribed gene (DTG); Fig. 1B]. Conversely, genes that have significant changes in RPFs independent of changes in mRNA counts are considered DTEGs (Fig. 1C).

A gene can be regulated transcriptionally and/or translationally, resulting in several different regulatory profiles. For example, if a gene is not a DTG, but is a DTEG, then it is exclusively regulated at the translational level. On the contrary, if a gene is both a DTG and DTEG, it is categorized as translationally intensified or buffered depending on the direction of the regulation (see Figure 1D, E for details).

There are a number of existing approaches to detect DTEGs by combining Ribo‐seq and RNA‐seq data, with the earliest report based on differences in TE (Ingolia et al., 2009). However, this approach does not take into account the variance, low expression of RPFs or mRNA counts, or batch effects, severely compromising the accuracy of detection. Several other approaches to detect DTEGs by modeling changes in TE have been developed subsequently: Ribodiff (Zhong et al., 2017), Xtail (Xiao, Zou, Liu, & Yang, 2016), Riborex (Li, Wang, Uren, Penalva, & Smith, 2017), and Anota2Seq (Oertlin et al., 2019). At their core, all of these approaches either utilize existing differential expression programs [e.g., DEseq2 (Love, Huber, & Anders, 2014) or EdgeR (Robinson, McCarthy, & Smyth, 2010)], or apply similar statistical assumptions to model the data. Unfortunately, these methods mostly miss essential functionalities of the underlying tools, vastly reducing their effectiveness. For instance, none of these methods, with the exception of Anota2Seq, allow for complex experimental design (i.e., with more than two conditions) or the use of alternative statistical setups (such as likelihood ratio tests for comparisons across time). Crucially, they do not account for the widespread batch effects in next‐generation sequencing datasets. Although stand‐alone tools for batch correction of sequencing data exist (Leek et al., 2010), differential expression tools require raw read counts to accurately model sample‐to‐sample variation (Anders et al., 2013; also see Table 1).

**Table 1:** Summary of Functionalities of Published Tools for Detection of Translational Regulation

Tool	Xtail	RiboDiff	RiboRex	Anota2Seq	ΔTE (our approach)
Sample‐to‐sample variance	Yes	Yes	Yes	Yes	Yes
Based on established statistical frameworks partly/or completely	Yes		Yes		Yes
Allows for complicated experimental design				Yes	Yes
Allows for covariates like batch effects				Yes	Yes
Classifies regulatory layers				Yes, but not all	Yes
Runtime (for primary human fibroblast dataset (Chothani et al., 2019), four pair‐wise comparisons)	∼120 min	∼60 min	∼5 min	∼20 min	∼5 min

This article outlines detection of DTEGs by introducing an interaction term into the statistical model of DESeq2, an approach that we refer to as ΔTE. We show that the fold change of the interaction term is equivalent to changes in TE, which detect DTEGs more accurately compared to all existing methods. When combining RNA‐seq and Ribo‐seq from two conditions, the interaction term can be used to model condition (untreated/treated) and sequencing methodology (Ribo‐seq/RNA‐seq). This allows the identification of significant differences between conditions that are discordant between sequencing methodologies. In order to do this, we design our generalized linear model with three components: the condition (c), the sequencing type (s), and an interaction term containing both (c:s); refer to the Commentary for details. The result is a ΔTE fold change and an associated false discovery rate (FDR) for significant changes of this fold change, which quantify the extent of translational regulation between conditions.

The protocols require the installation of R and basic familiarity with R or a Unix‐like environment. The workflow in the Basic Protocol includes a script, DTEG.R, which can be run in one step. This script implements two processes: (a) detection of DTEGs and (b) classification of genes into regulatory classes. An Alternate Protocol is included that carries out the same functions step‐by‐step in R, allowing flexibility in the case of complex experimental designs. Lastly, a Support Protocol is provided that outlines the workflow of obtaining count matrices from raw sequencing files, including a quality check of the data.

The Basic Protocol and example results are provided in our github repository: https://github.com/SGDDNB/translational_regulation.git.

## STRATEGIC PLANNING

Ribo‐seq can be carried out as described in the Current Protocols article Ingolia, Brar, Rouskin, McGeachy, & Weissman (2013). Similar to RNA‐seq analysis, careful experimental design is crucial. At least three biological replicates per condition or group are recommended for robust analysis of differential transcription, translation, and translational efficiency. The sample processing and library preparation should be carried out together for different conditions and sequenced on the same lane of a sequencing machine, or in a randomized order across lanes, to avoid batch effects. It is not possible to account for batch effects that are completely confounded by any other covariate. For instance, if all the control samples were prepared in one batch and the treatment samples in another batch, it would not be possible to distinguish differences due to treatment versus control from differences arising due to separate preparation batches. Thus, it is recommended to prepare control and treatment samples together. Alternatively, when there are large sample sizes, it is important to split the samples in such a way that the conditions are randomized. Samples should be sequenced to sufficient depth both in RNA‐seq and Ribo‐seq. Despite the presence of an experimental step to remove ribosomal RNA (rRNA) fragments from the input RNA, sequenced Ribo‐seq reads still include a fraction of rRNA sequences, which should be discarded before ΔTE analysis. Thus, it is recommended to sequence at least 20 million reads per sample. Single‐end 50‐bp read sequencing is sufficient, since ribosome footprints are expected to be 29 bp in length. After sequencing and processing the data, the fastq and alignment files should be checked for several quality measures, as described in the Support Protocol.

## ONE‐STEP DETECTION AND CLASSIFICATION OF DIFFERENTIAL TRANSLATION EFFICIENCY GENES (DTEG) USING DTEG.R

The RNA‐seq and Ribo‐seq data should be processed first as described in the Support Protocol, in order to determine translationally regulated genes. In the following steps, we quantify the change in TE of each gene, calculate an FDR value for this change, and categorize genes into regulation classes using the ΔTE approach. A DTEG is determined based on significant change in TE (FDR < 0.05). This protocol describes a wrapper script, DTEG.R, to detect and classify DTEGs. It also includes a script to visualize the transcriptional, translational, and TE changes for a gene of interest. Alternatively, the protocol can also be carried out step‐by‐step in R, allowing flexibility for complex experimental designs (see Alternate Protocol).

### Materials

#### Hardware

Computer running Unix, Linux or Mac OS X
Administrative privileges and internet connection to install packages

#### Software

DTEG.R and goi_viz.R script: These scripts can be downloaded from our github page by typing the following command in the terminal window:
$ git clone https://github.com/SGDDNB/translational_regulation.git
R: https://cran.r‐project.org/bin/windows/base/
Rstudio: https://www.rstudio.com/products/rstudio/download/
DESeq2: https://bioconductor.org/packages/release/bioc/html/DESeq2.html
DESeq2 can also be installed in R by typing the following command:
> if(!requireNamespace("BiocManager", quietly = TRUE)) install.packages("BiocManager")
> BiocManager::install("DESeq2")

#### Input files

ribo_counts.txt: RPF count matrix including genes as rows and samples as columns
rna_counts.txt: mRNA count matrix including genes as rows and samples as columns
sample_info.txt: Sample‐wise information about experimental condition, type of sample (RNA‐ or Ribo‐seq), and sample batch where applicable

### Preparing input files for DTEG.R

1 Generate read count matrices for both Ribo‐seq and RNA‐seq, as described in the Support Protocol.
  These files contain raw read counts obtained from read‐counting tools and should not be normalized or batch corrected. Each row represents a gene and each column represents a sample as shown below:

**Table:** 


Gene ID	Sample 1	Sample 2	Sample 3	Sample 4
ENSG0000XX	1290	130	2	1000
ENSG0000XY	0	2	10	5
….	..	..	..	..
ENSG0000ZZ	0	2	10	5
ribo_counts.txt

**Table:** 


Gene ID	Sample 5	Sample 6	Sample 7	Sample 8
ENSG0000XX	4000	2000	200	1200
ENSG0000XY	10	20	0	40
….	..	..	..	..
ENSG0000ZZ	0	2	10	5
rna_counts.txt
2 Create a tab‐separated sample information file with rows as samples and columns as condition and sequencing methodology.
  This file contains sample information for samples in both ribo_counts.txt and rna_counts.txt. The sample IDs should be unique and exactly match the sample names in the count matrices. This file contains two columns, Condition (treatment) and SeqType (sequencing methodology). Additionally, if there is a known batch effect in the dataset, it can also be included as another column, as shown below. If your experiment has more than one batch covariate, it is recommended to use the Alternate Protocol.

**Table:** 


Sample ID	Condition	SeqType	Batch
Sample 1	1	RIBO	1
Sample 2	1	RIBO	2
Sample 3	2	RIBO	1
Sample 4	2	RIBO	2
Sample 5	1	RNA	1
Sample 6	1	RNA	2
Sample 7	2	RNA	1
Sample 8	2	RNA	2
sample_info.txt

### Detecting and categorizing differentially transcribed genes and differential translation efficiency genes

**Figure 2:**
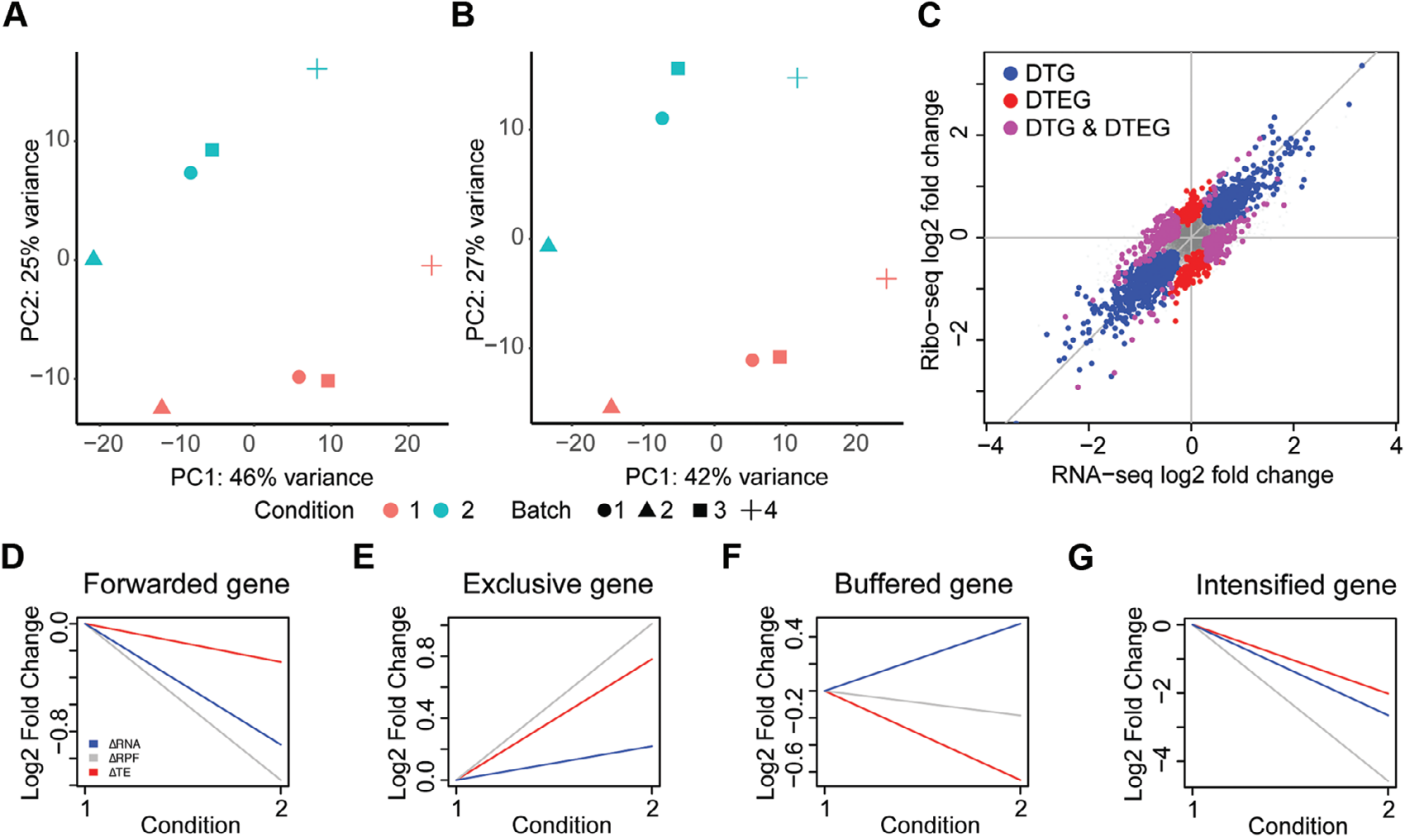
Translational regulation in sample data using DTEG.R script. Principal component analysis of (**A**) Ribo‐seq and (**B**) RNA‐seq datasets. (**C**) Scatter plot of log fold change values across both sequencing methodologies. Differentially transcribed genes (DTGs) and differential translation efficiency genes (DTEGs) are marked. (**D**‐**G**) Gene profiles of exemplars in each regulation class, translationally forwarded (D), exclusive (E), buffered (F), and intensified (G).

3 Open a Unix/Linux command line environment (“Terminal” application in a Linux operating system or Mac OS). Run script DTEG.R using the following command line:
$ Rscript DTEG.R arg1 arg2 arg3 arg4 arg5 arg6
where command arguments arg1‐6 are as follows:
Argument 1 (arg1): Ribo‐seq count matrix file path
Argument 2 (arg2): RNA‐seq count matrix file path
Argument 3 (arg3): Sample information file path
Argument 4 (arg4): Batch effect covariate: yes=1, or no=0
Argument 5 (arg5): Save Rdata file as a record for future use (optional, Default = 1)
Argument 6 (arg6): Verbose mode (optional, Default = 0)
  Example:
$ Rscript DTEG.R ./ribo_counts.txt ./rna_counts.txt ./sample_info.txt 1
  This command creates a Results/ directory including fold changes, gene lists for each regulatory group, and visualizations, as shown in Figure 2A‐G. For further details on the different output files created, refer to Understanding Results.

### Visualizing changes in mRNA counts, RPFs, and TE for a gene of interest

4 Run goi_vis.R.
  This step includes a one‐step script to visualize the fold changes across the condition given in the study for a gene of interest as shown in Figure 2D‐G:
$ Rscript goi_viz.R arg1 arg2 arg3 arg4
where command arguments arg1‐6 are as follows:
Argument 1 (arg1): Ribo‐seq fold change file path.
Argument 2 (arg2): RNA‐seq fold change file path.
Argument 3 (arg3): TE fold change file path.
Argument 4 (arg4): ENSEMBL gene ID
  The fold change files (arg1, arg2, and arg3) are generated in step 3 and are located in the results directory within the fold_changes subdirectory. ENSEMBL gene ids for your gene of interest can be obtained from https://www.ensembl.org/index.html. It is required to use the same genome version as used for obtaining the count matrix by Support Protocol.
  Example:
$ Rscript goi_viz.R
path/to/Results/directory/fold_changes/deltaRibo.txt
path/to/Results/directory/fold_changes/deltaRNA.txt
path/to/Results/directory/fold_changes/deltaTE.txt
ENSG00000095752
  This script is also part of the github directory and is automatically downloaded with the git clone command described in Materials, Hardware, above. Running this script saves an output file in the current directory (gene_id.pdf). This file saves a visualization of the ΔRPF, ΔmRNA, and ΔTE for the gene of interest. A line plot is used to show fold changes of the mRNA, RPF, and TE for the gene of interest across conditions as shown in Figure 2D‐G.

## STEP‐WISE DETECTION AND CLASSIFICATION OF DIFFERENTIAL TRANSLATION EFFICIENCY GENES USING R

This protocol performs the same task as the Basic Protocol, but step‐wise in R, describing each step allowing flexibility to users for complex experimental designs.

### Materials

#### Hardware

Computer running Unix, Linux or Mac OS X
Administrative privileges and internet connection to install packages

#### Software

R: https://cran.r‐project.org/bin/windows/base/
Rstudio: https://www.rstudio.com/products/rstudio/download/
DESeq2: https://bioconductor.org/packages/release/bioc/html/DESeq2.html
DESeq2 can also be installed in R by typing the following command:
> if(!requireNamespace("BiocManager", quietly =TRUE)) install.packages("BiocManager")
> BiocManager::install("DESeq2")

#### Input files

ribo_counts.txt: RPF count matrix including genes as rows and samples as columns
rna_counts.txt: mRNA count matrix including genes as rows and samples as columns
sample_info.txt: Sample‐wise information on sequencing methodology used, condition and batch

1 Prepare input files as described in steps 1 and 2 of the Basic Protocol.
  Additionally, using this protocol, the sample information file can have more columns for other covariates that can be included in the model design, as described in step 3.
2 Open Rstudio and load count matrices and sample information file:
> ribo_counts = read.delim(“ribo_counts.txt”)
> rna_counts = read.delim(“rna_counts.txt”)
> sample_info = read.delim(“sample_info.txt”)
  These commands assume that all required files are within your working directory. In case they are not, provide the full path to the input file in the read.delim command.
3 Create DESeq2 object for the combined dataset of Ribo‐seq and RNA‐seq counts. The interaction term should be included in the linear model design as follows:
> ddsMat = DESeqDataSetFromMatrix(
countData=cbind(ribo_counts,rna_counts),
colData=sample_info,
design=∼ Condition+SeqType+Condition:SeqType)
  The data can be tested for batch effects using principal component analysis (PCA). If there is a batch effect/other covariate, the design can be modified by adding the covariate to the design as: ∼ Batch + Condition + SeqType + Condition:SeqType.
4 Run DESeq2:
> ddsMat = DESeq(ddsMat)
  This step carries out estimation of size factors, estimation of dispersion, and model fitting. The relevel function in R can be used prior to running DESeq2 to assign a reference level from which comparisons will be made. It is important that the reference level for sequencing type be RNA‐seq; the reference level for condition can be selected based on the experiment.
5 Obtain fold changes for TE:
> res = results(ddsMat, name=“Condition2.SeqTypeRIBO”)
  This step calculates the gene‐wise fold change and its statistical significance for a given comparison. DESeq2 calculates this change between different groups that are described in the sample information file and model design. The calculated comparisons can be obtained by using resultsNames(ddsMat). For instance, name=“Condition_2_vs_Condition_1” quantifies changes between condition 2 and condition 1 using the reference level RNA‐seq (see step 4). Similarly, name=“Sequencing_Ribo_vs_RNA” quantifies the difference between Ribo‐seq counts and RNA‐seq counts using the reference level as condition 1. These can also be supplied using the contrast parameter instead of the name parameter as follows: contrast=c(“Condition”,“2”,“1”) and contrast=c(“SeqType”,“RIBO”,“RNA”), respectively. For interaction term fold change we use *name=*“*Condition2.SeqTypeRIBO*”. This quantifies the change in TE in condition 2 versus baseline condition 1. Refer to Commentary for the mathematical proof that the interaction coefficient is equivalent to TE.

### Detecting differential translation efficiency genes

6 Store the list of DTEGs in a file:
> write.table(res[which(res$padj<0.05), ], “DTEGs.txt”, quote=F)
  DTEGs are genes which have a significant interaction term fold change. FDR values can be chosen based on user preference; here we recommend using FDR < 0.05.
7 Run DESeq2 for mRNA counts in order to obtain DTGs:
> ddsMat_rna = DESeqDataSetFromMatrix(
countData=rna_counts,
colData=sample_info[which(samples_info$SeqType == “RNA”),],
design=∼Condition)
> ddsMat_rna = DESeq(ddsMat_rna)
> res_rna = results(ddsMat_rna, name="Condition_2_vs_1")
> res_rna = lfcShrink(ddsMat_rna,name="Condition_2_vs_1",res=res_rna)
> write.table(res_rna[which(res_rna$padj<0.05), ],“DTGs.txt”, quote=F)
  DTGs are genes that have a significant change in the mRNA counts. To obtain DTGs, we run DESeq2 separately for mRNA counts and use the same FDR as above (FDR < 0.05). These data may also be tested for batch effects using PCA, and if any batch effects are identified, they should be included in the sample_info.txt file and in the design as ∼Condition + Batch.

### Categorizing genes into different regulation groups

8 Run DESeq2 for RPFs (Ribo‐seq counts):
> ddsMat_ribo = DESeqDataSetFromMatrix(
countData=ribo_counts,
colData=sample_info[which(samples_info$SeqType == “RIBO”),],
design=∼Condition)
> ddsMat_ribo = DESeq(ddsMat_ribo)
> res_ribo = results(ddsMat_ribo,name="Condition_2_vs_1")
> res_ribo =
lfcShrink(ddsMat_ribo,name="Condition_2_vs_1"),res=res_ribo)
  In order to classify genes into different regulation classes, quantification of the change in the RPFs is required. Similar to mRNA counts, these data should also be tested for batch effects, and, if any batch effects are identified, the batches should be included in the file sample_info.txt and the model design.
9 Obtain genes for each regulation class described in Figure 1D, E.
  For each gene, the change in RPFs (ΔRPF), change in mRNA counts (ΔRNA), and change in its TE (ΔTE) are combined to determine its regulation group, as shown in Table 2. It is recommended to use an FDR threshold of 0.01 or 0.05.
  - *Forwarded*: Genes driven by transcriptional regulation. These genes do not have a change in TE, and the change in RNA drives the change in RPFs. Hence, genes that have significant ΔRPF and ΔRNA but that do not have a significant ΔTE fall into this class.
> forwarded = rownames(res)[which(res$padj > 0.05 & res_ribo$padj < 0.05 & res_rna$padj < 0.05)]
  - *Exclusive*: Genes regulated exclusively by translation. This means that the change in TE is driven by change in RPFs exclusively, and there is no change in mRNA counts. Hence, genes with significant ΔTE and ΔRPFs but no significant change in mRNA counts belong to this group.
> exclusive = rownames(res)[which(res$padj < 0.05 & res_ribo$padj < 0.05 & res_rna$padj > 0.05)]
  - *Intensified and buffered*: Genes regulated both by transcriptional and translational regulation (significant ΔRNA, ΔRPFs, and ΔTE) include intensified and buffered genes. These genes are both DTGs and DTEGs.
> both = rownames(res)[which(res$padj < 0.05 & res_ribo$padj < 0.05 & res_rna$padj < 0.05)]
  In order to further categorize these genes into intensified and buffered genes, the direction of the transcriptional change (ΔRNA) and translational efficiency change (ΔTE) are compared.
*Intensified*: Genes for which the translational regulation acts with the transcriptional regulation change. These genes have the translational change in the same direction as their transcriptional change:
> intensified = rownames(res)[both[which(res[both,2]*res_rna[both,2] > 0)]]
*Buffered*: Genes for which the translational regulation counteracts the transcriptional regulation change. In these genes, the transcriptional change (ΔRNA) and translational efficiency change (ΔTE) are in the opposite direction:
> buffered = rownames(res)[both[which(res[both,2]*res_rna[both,2] < 0)]]
  There is also a special case of buffered genes wherein the transcriptional change is cancelled out by the change in TE to the point of no significant change in RPFs. Hence, genes with significant ΔTE and ΔRNA but that do not have a significant ΔRPF are also considered as translationally buffered.
> buffered = c(rownames(res)[which(res$padj < 0.05 & res_ribo$padj > 0.05 & res_rna$padj < 0.05)], buffered)

**Table 2:** Classification of Genes into Regulatory Classes Shown in Figure 1D^a^^,^ b

Class	ΔRPF	ΔRNA	ΔTE	Fold change direction	DTG/DTEG	Schematic
No change	n.s.	n.s.	n.s.	No change at either regulatory levels	None	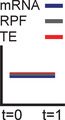
Forwarded	sig	sig	n.s.	Change in RPF is in the same direction as change in RNA	DTG	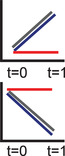
Exclusive	sig	n.s.	sig	Change in RPF is not driven by change in RNA	DTEG	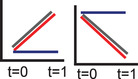
Intensified	sig	sig	sig	Change in TE is counteracting the change in RNA	DTG and DTEG	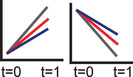
Buffered	sig	sig	sig	Change in TE is completely counteracting the change in RNA; No change in RPF	DTG and DTEG	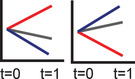
Buffered (special case)	n.s.	sig	sig	Change in TE is intensifying change in RNA	DTG and DTEG	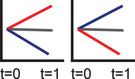

a DTG, differentially transcribed gene; DTEG, differential translation efficiency gene; n.s., not significant; sig, significant.

b Genes with any other combinations, i.e., (1) RPF: n.s.; RNA: sig, TE: n.s.; (2) RPF: sig; RNA: n.s., TE: n.s.; and (3) RPF: n.s.; RNA: n.s., TE: sig; are considered as undetermined as they cannot be grouped into any of the classes.

10 Visualize the global translational and transcriptional regulation as in Figure 1E.
> max_val = max(res_ribo[,2],res_rna[,2],na.rm = T)
> plot(y=res_ribo[,2],x=res_rna[,2],
xlab="RNA‐seq log2 fold change",
ylab = "Ribo‐seq log2 fold change", asp=1, pch=16,
col=rgb(128/255,128/255,128/255,0.1), ylim=c(‐
max_val,max_val), xlim=c(‐max_val,max_val),cex=0.4)
> abline(a=0,b=1,col="gray")
> abline(h=0,v=0,col="gray")
> points(y=res_ribo[forwarded,2], x=res_rna[forwarded,2],
pch=16,col=rgb(0,0,1,1))
> points(y=res_ribo[exclusive,2], x=res_rna[exclusive,2],
pch=16,col=rgb(1,0,0,1))
> points(y=res_ribo[intensified,2], x=res_rna[intensified,2],
pch=16,col=rgb(1,0,1,1))
> points(y=res_ribo[buffered,2], x=res_rna[buffered,2],
pch=16,col=rgb(1,0,1,1))
  These steps plot the global fold changes of mRNA counts versus the RPFs, as shown in Figure 1E. Refer to Understanding Results for more details.

### Visualizing changes in mRNA counts, RPFs, and TE for a gene of interest

11 Visualize the transcriptional, translational, and TE changes of a given gene id [id] using a line plot.
> ymax=max(res_ribo[**id**,2],res_rna[**id**,2],res[**id**,2],0)
> ymin=min(res_ribo[**id**,2],res_rna[**id**,2],res[**id**,2],0)
> plot(c(0,1), c(0,res_ribo[**id**,2]), type="l",col="gray",
ylim=c(ymin,ymax), ylab="Log2 fold change",xlab="",xaxt="n")
> lines(c(0,1), c(0,res_rna[**id**,2]),type="l",col="blue")
> lines(c(0,1), c(0,res[**id**,2]), type="l",col="red")
> legend("bottomleft",c("RNA","Ribo","TE"),fill=c("blue","gray","red"),
cex=1, border = NA, bty="n")
> axis(1,at=c(0,1),labels=c(1,2),las=1)
  This step carries out the same function as step 4 of the Basic Protocol. It requires a gene id for your gene of interest, which can be obtained from https://www.ensembl.org/index.html, or can be based on the genome annotation file used to obtain count matrices with Support Protocol. The input **id** should be a row name in the count matrix file.

## WORKFLOW FROM RAW DATA TO READ COUNTS

The raw sequencing data should be processed prior to the Basic Protocol or Alternate Protocol, as shown below. It is also strongly recommended to carry out quality check for the raw and processed data as described in the following steps.

### Materials

#### Hardware

Computer running Unix, Linux or Mac OS X

#### Software

Trimmomatic: http://www.usadellab.org/cms/?page=trimmomatic
Bowtie2: http://bowtie‐bio.sourceforge.net/bowtie2/index.shtml
STAR: https://github.com/alexdobin/STAR
subread: http://subread.sourceforge.net/
FastQC: https://www.bioinformatics.babraham.ac.uk/projects/download.html
MultiQC: https://multiqc.info
Ribo‐TISH: https://github.com/zhpn1024/ribotish/blob/master/INSTALL.rst

#### Input files

seq.fastq.gz: Raw sequencing files for both Ribo‐seq and RNA‐seq
adaptors.fa: List of adaptor sequences in a fasta format
abundant.fa: List of abundant sequences (rRNA, transfer RNA (tRNA), and mitochondrial RNA (mtRNA)) in fasta format
organism.fa: Genome sequence in fasta format for the organism used in the study
organism.gtf: Genome‐wide transcript annotations in gene transfer format (GTF) for the organism used in the study

### Processing the raw sequencing data to generate gene expression count matrix files

1 Trim adaptor sequences from reads:
$ java ‐jar trimmomatic‐0.36.jar SE ‐phred33 seq.fastq.gz outfile ILLUMINACLIP:adaptors.fa:2:30:10 MAXINFO:20:0.5 MINLEN:20
  where:
seq.fastq.gz is the raw sequencing file;
outfile is the output file prefix;
adaptors.fa is the list of sequences of adaptors used for sequencing in fasta format;
MINLEN is the minimum length of reads required to retain.
  The arguments are based on Trimmomatic V0.36, and other parameters can be explored as described in the manual, which can be obtained from http://www.usadellab.org/cms/?page=trimmomatic. The minimum length required is set to 20, as the expected read length for RPFs is 29. This command trims the adaptor sequences from raw read sequences and saves an output file (outfile.fastq.gz) which is used as input file for step 2.
2 Remove reads mapping to abundant sequences.
  This step first prepares a bowtie2 index for the known abundant sequences: rRNA, tRNA, and mtRNA. These sequences are considered contaminants of Ribo‐seq data, since we want to capture only RPFs. Therefore, reads mapping to these contaminant sequences are removed prior to further analysis:
$ bowtie2‐build abundant.fa index
  Where:
abundant.fa is the list of abundant sequences (rRNA, tRNA, and mtRNA) in fasta format;
index is the prefix for the bowtie index output files.
$ bowtie2 ‐L 20 ‐x index ‐‐un‐gz outfile ‐U infile ‐S samfile
  Where:
infile is the trimmed sequencing fastq.gz file, which was the outfile obtained in step 1;
outfile is the output filename for unmapped reads in fastq.gz format;
samfile is the output filename for mapped reads in SAM format;
index is the prefix used for the bowtie index.
  The arguments are based on Bowtie2 (V2.2.9), and other parameters can be explored as described in the manual. This function builds the index for abundant sequences, aligns the reads to the same, and saves a fastq.gz file, retaining only the unmapped reads. This output fastq.gz file comprises a cleaned set of reads that do not map to the abundant sequences and represent the RPFs. The reads in this file are further mapped to the genome in the next step.
3 Align reads to the genome file using the transcriptome index.
  Before aligning the reads, it is required to generate a transcriptome index for the organism of interest. The required input files, the genome fasta and annotation files, can be downloaded from the Ensembl database at https://asia.ensembl.org/info/data/ftp/index.html. These files should be for the same organism and same genome build. Run the following commands to generate the index, followed by alignment of reads to the same:
$ STAR ‐‐runMode genomeGenerate ‐‐genomeDir ‐‐genomeFastaFiles organism.fa ‐‐sjdbGTFfile organism.gtf
  Where:
organism.fa is the genome sequence in fasta format;
organism.gtf is the genome‐wide transcript information;
genomeDir is the directory name for the output STAR index files.
$ STAR ‐‐runThreadN 16 ‐‐alignSJDBoverhangMin 1 ‐‐alignSJoverhangMin 51 ‐‐outFilterMismatchNmax 2 ‐‐alignEndsType EndToEnd ‐‐genomeDir star2.5.2b_genome_index ‐‐readFilesIn infile ‐‐readFilesCommand gunzip ‐c ‐‐outFileNamePrefix outPrefix ‐‐quantMode GeneCounts ‐‐outSAMtype BAM SortedByCoordinate ‐‐limitBAMsortRAM 31532137230 ‐‐outSAMattributes All
  Where:
genomeDir is the directory name for the STAR index files generated in the previous step;
infile is the cleaned fastq.gz file, which was the outfile in step 2;
outPrefix is the prefix for the output filenames.
  The arguments are based on STAR version 2.5, and other parameters can be explored as described in the manual. This function builds a STAR index for a given fasta and GTF, aligns the reads to the same, and saves an alignment file in the BAM format.
4 Count reads mapped to coding regions of genes:
$ featureCounts ‐t CDS ‐g gene_id ‐O ‐s 1 ‐J ‐R ‐G organism.fa ‐a organism.gtf ‐o outfile infile_path/*bam
  Where:
organism.fa is the genome sequence in fasta format;
organism.gtf is the genome‐wide transcript information;
outfile is the output file name for the count matrix;
infile_path is the path to the directory containing all bam files obtained in step 3.
  The arguments are based on FeatureCounts V1.5.1, and other parameters can be explored as described in the manual. This function counts the reads that have mapped to a given region and summarizes gene‐wise counts for each alignment file. This script requires all bam files to be in one directory to make a combined count matrix for all files. Alternatively, this command can be run for each .bam file generated in step 3, and then the individual count files can be combined into one matrix prior to the Basic Protocol or Alternate Protocol.

### Quality check of the raw sequencing data and processed files

**Figure 3:**
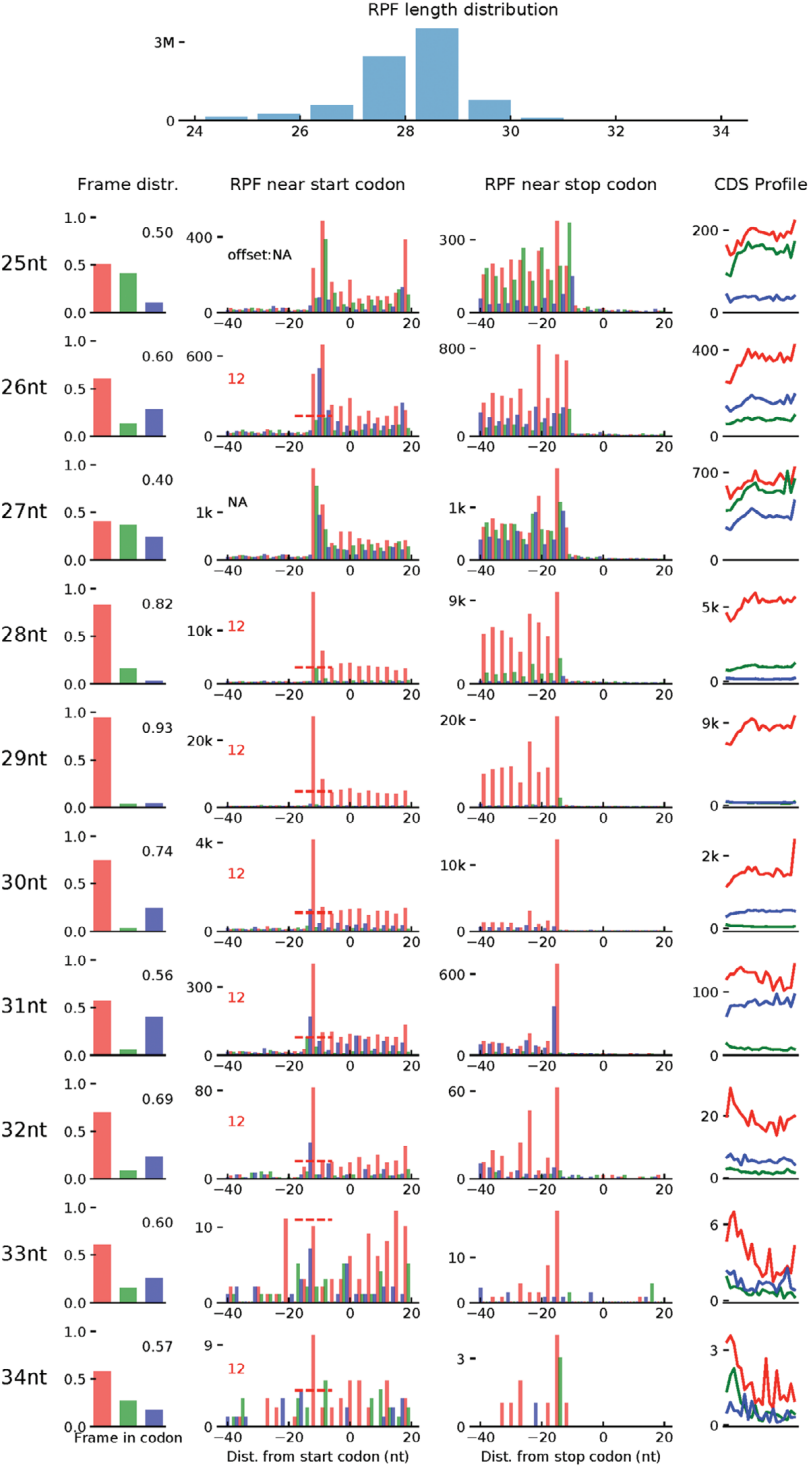
Quality check of Ribo‐seq data using Ribo‐TISH. The tool RiboTISH provides several visualizations to investigate the data quality of Ribo‐seq. First, it includes the length distribution for the Ribo‐seq reads as a histogram. As the length of ribosome‐protected mRNA fragment (RPF) is expected to be around 29 base pairs, the length distribution of the sequenced reads is used as a quality measure. Second, the 3‐nucleotide periodicity of the RPFs mapped on all known protein‐coding genes is shown for each read length. As shown, in these data, we have a high (93%) percentage of reads in Frame 1 with the predominant read length (29 bp). This is shown using a histogram of read coverage in the three frames, a barplot of the number of RPFs in each position around the START codon and STOP codon, and lastly a density plot for read coverage on the coding sequence across all genes.

5 Run FastQC:
$ fastqc [filename].fastq.gz
  The user needs to replace [filename] with the name of the raw sequencing or trimmed files. This command saves an .html file that documents the sequencing data quality. This includes sample‐wise read quality, %GC content, adaptor content, over‐represented sequences in the reads, read length distribution, etc. Refer to the Resources for the FastQC manual, which includes more details.
6 Run MultiQC to summarize QC for all the steps in Support Protocol:
$ multiqc/path/to/parent/directory/of/all/log/files/
  The path to the parent directory for results from steps 1 to 4 needs to be provided as an argument to MultiQC. This command saves an .html file which summarizes the sequencing quality, trimming results, abundant sequence removal, mapping, and read counting results for all samples together. Refer to the Resources for link to the MultiQC website.
7 Calculate and visualize periodicity of Ribo‐seq dataset:
$ samtools index [bam_file_prefix].bam
$ ribotish quality ‐b [bam_file_prefix].bam ‐g ensemble.gtf
  The first step creates an index for the alignment file (.bam) generated in step 3. The user should replace [bam_file_prefix] with the outfile prefix specified in step 3 for alignment files. The second step evaluates the quality of the alignment file. This step saves a .pdf that shows the read‐length distribution and periodicity of the Ribo‐seq data, as shown in Figure 3.

## COMMENTARY

### Background Information

Several methods have been developed for read alignment and read counting since the advent of RNA‐seq (see Current Protocols article Ji & Sadreyev, 2018). In the Support Protocol, we use STAR, bowtie2, and feature counts for both Ribo‐seq and RNA‐seq datasets. These tools can be chosen based on user preferences. Due to the slightly different nature of Ribo‐seq reads, it is important to modify parameters accordingly. For instance, since the RPFs are expected to be around 29 bp, soft clipping of reads can be quite detrimental to alignment pipelines and is not recommended. Furthermore, RNA‐seq pipelines use six to eight allowed mismatches, but this can be quite large in a 29‐bp read. We recommend one to two allowed mismatches for a robust downstream analysis.

In this protocol, we describe an interaction term–based TE analysis using DESeq2, but a similar model can also be incorporated in other generalized linear model–based differential expression tools such as edgeR. Previously, several publications have used DESeq2 to identify DTEGs, but in a suboptimal manner. For instance, these tools are used to calculate ΔRPF and ΔRNA, following which changes in TE are calculated using the ratio ΔRPF/ΔRNA. The translationally regulated genes are then identified using |*z*‐score| > 1.5 (Xu et al., 2017). This approach is referred to as the Ratio method in the benchmarking analyses. Another approach used previously also involves quantification of ΔRPF and ΔRNA using DESeq2. However, in this case, the translationally regulated genes are defined as genes with significant changes in either RPF or mRNA levels, but not both (Schafer et al., 2015). This approach falsely calls genes as translationally exclusive or buffered in cases where counts have a large variance across samples or are very low in either sequencing methodology. It would be unable to differentiate between a case where a gene is translationally regulated and a case where a gene has low counts/high variation in one of the sequencing methodologies. This is referred to as the Overlap method in the benchmarking analyses.

In order to benchmark the performance of our approach, we use three independent simulation datasets, two derived from previous publications (Oertlin et al., 2019, Xiao et al., 2016) and a third that was newly generated to evaluate the performance of the tools in the presence of a batch effect. Despite DESeq2 being a key component of many existing approaches, it was either not included or not used correctly in previous benchmarks.

**Figure 4:**
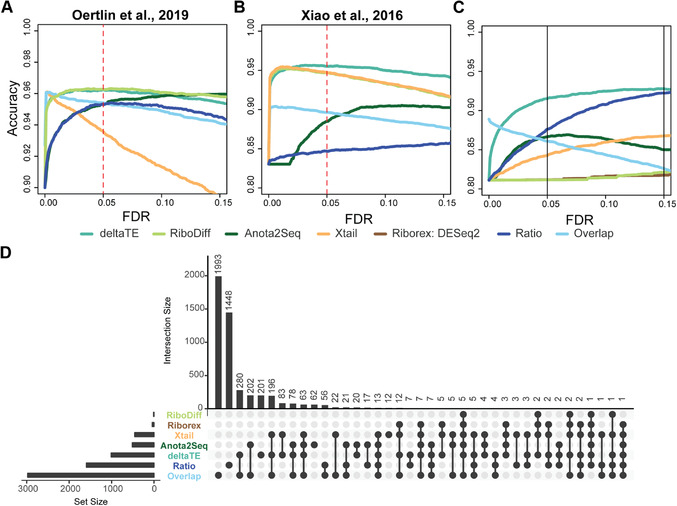
Benchmarking of published tools to detect differential translation efficiency genes (DTEGs). Simulation datasets (**A**) derived from Oertlin et al. (2019), (**B**) derived from Xiao et al. (2016), and (**C**) generated using the Polyester package to introduce batch effects were used. All three simulations show that ΔTE outperforms all other published methods. Comparisons are made using all the DTEGs as the true set. Since Anota2Seq has two different functions for obtaining exclusive and buffered genes, the results are combined prior to comparison. Riborex is omitted in simulated datasets without batch effects (A, B), since it is equivalent to the ΔTE approach in these cases. The ratio method is based on quantifying the ratio of DESeq2 fold changes for mRNA counts and RPF. The overlap method identifies DTEGs as genes which have either significantly changing mRNA counts or RPFs but not both. (**D**) Analysis on published data showed inability of previous tools to reliably identify DTEGs.

Figure 4A‐C shows accuracy curves for detection of DTEGs in each of these benchmarking datasets across typically used FDR thresholds. A full receiver operating characteristic (ROC) and area under the curve (AUC) analysis can be found in the associated web resource. Our benchmarking shows that ΔTE has a superior accuracy in comparison to existing methods, especially in the presence of a batch effect. The only method that performs at a similar level to ΔTE is RiboDiff, in the case of the data from Oertlin et al. (2019) (Fig. 4A). However, in the presence of a batch effect or based on the data from Xiao et al. (2016), ΔTE is superior.

To further verify that this effect is not confined to simulated data, we analyzed RNA‐seq and Ribo‐seq data derived from our recent study on cardiac fibrosis (Chothani et al., 2019). This experiment contained cardiac fibroblasts from four different individuals, and, as a result, has a pronounced patient‐related batch effect accounting for roughly 25% of the variance within the data. While it is not possible to quantify the accuracy of these real data, it is consistent with the benchmark results. For instance, the overlap and ratio methods predict the highest number of DTEGs, but were shown to have high FP rates in the benchmarking. Conversely, other existing tools that detect very few genes consistently showed the worst accuracy in the benchmark containing batch effects.

Taken together, the three benchmark studies and real data analysis strongly suggest that the ΔTE method is the most suitable for any integrative analysis of Ribo‐seq and RNA‐seq data, being both accurate and robust regardless of the data being analyzed.

### Critical Parameters and Troubleshooting

Experimental design is one of the most important factors for efficient detection of DTEGs. In best‐case scenarios, designs should avoid batch effects. Unavoidable batch effects should not be completely confounded with the groups of interest. This would lead to a non‐full‐rank design in DESeq2, which makes correction of the batch effect impossible within the model. It is recommended to evaluate samples for batch effects or outliers using PCA prior to analysis. Batch effects can be checked by visualizing PC1 and PC2, which account for most variance, and the remaining PCs can also be explored to identify minor batch effects.

Installation of tools can be quite cumbersome due to different platforms and versions. Apart from the standard installation procedures provided in the protocols, the required tools can also be installed using the Anaconda software package https://docs.anaconda.com/anaconda/install/. For instance, some of the tools used in Support Protocol can be installed with the following commands:

conda install ‐c bioconda trimmomatic
conda install ‐c bioconda bowtie2
conda install ‐c bioconda subread
conda install ‐c bioconda star

### Statistical Analysis

DESeq2 utilizes the Wald test for differential expression analysis in pair‐wise data (i.e., two conditions). If the experimental design includes a time‐series, each time point can be compared pair‐wise using the Wald test. Alternatively, the likelihood ratio test within DESeq2 can be used, which is more suitable to identify differences across a time‐series.

#### Mathematical proof: Interaction term coefficient is equivalent to the changes in translation efficiency

The interaction term in a generalized linear model provides a coefficient that models the non‐additive effects of two variables. The design described in the protocol corresponds to the following linear equation (Equation 1):

(1) log  count sc,s=β0+β1c+β2s+β3c×s

where c = condition and s = sequencing methodology. When this is used to model changes in the gene expression between conditions, it is possible to disentangle the transcriptional and translational contributions. For example, in an experimental setup with Ribo‐seq (s = 1) and RNA‐seq (s = 0) carried out over two conditions (c = 0 or 1), the gene‐wise transcriptional and translational changes are calculated as follows.

First, the coefficients contributing towards the mRNA levels (s = 0) are identified for each condition (c = 0 or 1) separately. We then compute the difference of the identified coefficients to obtain the change in transcription.

mRNA levels given condition (c = 1) and sequencing methodology (s = 0) using Equation 1:

log  count sc=1,s=0=β0+β1×1+β2×0+β3×1×0=β0+β1

mRNA levels given condition (c = 0) and sequencing methodology (s = 0):

log  count sc=0,s=0=β0+β1×0+β2×0+β3×0×0=β0

Change in mRNA levels between the two conditions:

log count sc=1,s=0−log count sc=0,s=0=β0+β1−β0=β1= transcriptional  changes

Similarly, the coefficients contributing towards the RPF counts (s = 1) can be quantified, and the differences signify the change in RPFs of a gene across conditions.

RPFs given condition (c = 1) and sequencing methodology (s = 0) using Equation 1:

log  count sc=1,s=1=β0+β1×1+β2×1+β3×1×1=β0+β1+β2+β3

RPFs given condition (c = 0) and sequencing methodology (s = 0) using Equation 1.

log  count sc=0,s=1=β0+β1×0+β2×1+β3×0×1=β0+β2=β0+β2

Changes in RPFs between the two conditions:

log count sc=1,s=1−log count sc=0,s=1=β0+β1+β2+β3−(β0+β2)=β1+β3

In order to obtain the translational changes that are independent of transcriptional changes, we subtract the changes in mRNA from the change in RPFs. This is equivalent to the interaction term coefficient *β*_3_ as follows:

Changes in RPFs – Changes in mRNA levels:

$$\begin{array}{c} =\left(\beta_{1\;}+\beta_{3\;}\right)-\beta_{1\;}\;=\;\beta_{3\;} \end{array}$$

Thus, β_3_, which is the interaction term coefficient, is equal to translational changes that are independent of transcriptional changes. Importantly, it is also possible to show that this interaction term coefficient is equivalent to the fold change in TE:

β3=ChangesinRPFs−ChangesinmRNAlevels=[log( count sc=1,s=1)−log( count sc=0,s=1)]−[log( count sc=1,s=0)−log( count sc=0,s=0)]=[log( count sc=1,s=1)−log( count sc=1,s=0)]−[log( count sc=0,s=1)−log( count sc=0,s=0)]= log  count sc=1,s=1 count sc=1,s=0− log  count sc=0,s=1 count sc=0,s=0= log # RPFs  in  condition 1# mRNAs  in  condition 1− log # RPFs  in  condition 0# mRNAs  in  condition 0

Further, since TE is defined as the ratio of mean normalized Ribo‐seq counts (RPFs) over RNA‐seq counts,

β3=log( TE  in  condition 1)− log ( TE  in  condition 0)=Δ TE

where TE is the translation efficiency.

As a result, the fold change (and associated adjusted *p*‐value) obtained using the interaction term coefficient β_3_ describes, for each gene, the change in TE. Genes with a significant adjusted *p*‐value for ΔTE are considered as DTEGs. Since this is a linear model, the design can also be extended to facilitate more complex experimental designs, such as batch effects or other covariates, making it a powerful tool for identifying DTEGs.

### Understanding Results

The Basic Protocol implements the script DTEG.R from our github repository (https://github.com/SGDDNB/translational_regulation). This generates a results directory which includes two subdirectories (fold_changes/, gene_lists/) and one file (Results_figures.pdf).

In order to demonstrate the usage and output of DTEG.R, we utilized Ribo‐seq and RNA‐seq count data from our recent study (Chothani et al., 2019) on primary human fibroblasts stimulated with TGFB1. We obtained a subset of this dataset using four patients and two conditions (unstimulated, stimulated). The results directory generated after following the Basic Protocol on this dataset is also saved in the github repository.

The subdirectory fold_changes/ contains three files, namely: deltaRibo.txt, deltaRNA.txt, and deltaTE.txt. These files store gene‐wise expression changes across the given conditions in RPF, RNA, and TE, respectively. The results are obtained using DESeq2 and are saved in its standard output format. The two important columns, gene‐wise log fold changes and the associated adjusted *p*‐values, are used to determine gene expression changes between the two conditions. Generally, *p*_adj_ < 0.05 is used as a threshold for determining genes that are changing significantly. A threshold for the absolute log fold change can also be used to select only high‐effect sizes. The genes obtained using these thresholds are considered as significantly changing across the given condition or treatment. Genes passing these thresholds in deltaRNA.txt are those with a significant change in RNA and are considered DTGs, and genes passing these thresholds in deltaTE.txt are considered DTEGs.

Furthermore, the combination of changes in RPF, RNA, and TE are used to determine a gene's regulatory class, as shown in Figure 1D. A subdirectory, gene_lists/, contains files that list genes from each regulatory class. These include genes that have been identified as either DTG or DTEG and then further classified into translationally forwarded, buffered, exclusive, or intensified (see details in Table 2). Genes that are classified as forwarded are transcriptionally driven and exhibit no change in TE. On the contrary, translationally exclusive genes exhibit changes in TE but no change in transcription, which implies that these genes are only regulated translationally. Buffered and intensified genes have changes in TE as well as changes in RNA. If these changes in RNA counteract the change in TE, we consider them as translationally buffered, while if RNA changes act with changes TE, we consider them intensified. In each case, these genes are under both transcriptional and translational regulation.

Beyond what is described in these protocols, to understand the potential functions of the different gene regulatory classes, a gene set enrichment analysis (GSEA) or gene ontology (GO) overrepresentation analysis is recommended. Furthermore, hierarchical clustering of the gene‐wise fold changes can also be performed to identify subgroups of genes that have a similar regulatory profile.

Lastly, the script generates a file, Results_figures.pdf, which includes three main visualizations of (1) a PCA, (2) global fold changes, and (3) gene‐wise fold changes.

The PCA is conducted for both the Ribo‐seq and RNA‐seq count data. A PCA transforms the data in such a way that each component captures a different source of variation within the data, with the first component (PC1) capturing the largest source of variance in the data. Thus, a PCA can be used to determine any batch effect that is a source of variation in the data. In the example data, it shows that PC1 accounts for 42% of the variance in the Ribo‐seq and 46% of the variance in the RNA‐seq data. Importantly, PC1 separates the individual patients in both the datasets, indicating that the largest variance in these data is due to the difference between patients in the study. Since these datasets were generated to study the changes in different conditions (unstimulated/stimulated), it is important to remove this patient effect (Fig. 2A, B). Therefore, in this case, the DTEG.R script should be run with the batch effect parameter (Argument 4) set to 1.

The .pdf file also includes a visualization of the global fold changes, as shown in Figure 2C. This is drawn using a scatter plot of the fold changes in RNA and RPFs. The plot also highlights whether the gene is a DTG and/or a DTEG. This plot gives an overview of the overall impact of translational regulation in the system. As such, it can be used to determine the dominant mode of regulation in the dataset and visualize the overall effect sizes of the different regulation types. For instance, if there were very few DTEGs and many DTGs found, it would imply that there is very little translational regulation in the system, and most of the changes occur via transcriptional regulation.

In order to look at individual examples, the file further visualizes the gene‐wise fold changes for the genes with the strongest effect in each category (Fig. 2D‐G). A line plot is used for visualizing the changes from unstimulated to stimulated in this study. These line plots can be generated for any gene of interest using step 4 in the Basic Protocol or step 11 in the Alternate Protocol.

### Time Considerations

The protocol takes a couple of minutes on a standard computer for the example dataset, which includes four samples and two conditions. This could vary based on the number of samples and conditions to be tested.
